# Correction for Polarization Current Curve of Polymer Insulation Materials in Transformers Considering the Temperature and Moisture Effects

**DOI:** 10.3390/polym12010143

**Published:** 2020-01-06

**Authors:** Hanbo Zheng, Benhui Lai, Yiyi Zhang, Jiefeng Liu, Shichang Yang

**Affiliations:** College of Electrical Engineering, Guangxi University, Nanning 530004, China; hanbozheng@163.com (H.Z.); 15207186441@163.com (B.L.); liujiefeng9999@163.com (J.L.); ysc972@yeah.net (S.Y.)

**Keywords:** transformer polymer insulation, polarization and depolarization current (PDC), shift factor, moisture content, temperature correction

## Abstract

Depending on the study of the master curve technique, a temperature correction model for the polarization current of transformer polymer (cellulose) insulation, considering the effects of both moisture content (*mc*%) and temperature is proposed. In the current work, the shift factors of polarization current curves of samples with various moisture contents are extracted at different temperatures. Then, the variation law among the shift factor, test temperature, and moisture content are studied so as to establish the corresponding functional relationship. The findings reveal that the modified model derived from the above functional relationship could be employed to perform the temperature correction of oil-immersed polymer samples with various insulation states. Therefore, the proposed temperature correction model in this paper will promote the state assessment of the field transformer polymer insulation.

## 1. Introduction

The main insulation system of the oil-immersed power transformer is composed of liquid insulation and solid polymer (cellulose) insulation. In the context of the irreversible characteristics of the degradation of the solid polymer insulation, its insulation performance determines the overall service life of the transformer [1,2,3]. Further, the operation of the transformer is accompanied by a complex electric field and temperature field, which leads to an overall decline in the insulation properties of polymer materials, which further effects the normal operation of the entire power grid [4]. Therefore, the study of the state assessment of transformer polymer insulation materials remains significant.

In the last decades, the state assessment methods of transformer polymer insulation materials based on the traditional chemical and electrical technique have been widely reported [5,6,7]. However, traditional methods require further research in the field test due to various limitations. For example, typical chemical methods, such as dissolved gas analysis (DGA) and dissolved furfural in oil, are susceptible to the activity of oil filtering and replacement. Further, the insulation information carried by traditional electrical methods, such as insulation resistance and dielectric dissipation factor at 50 Hz, is relatively simple, and cannot fully characterize the information of transformer polymer insulation materials. In view of the above issues, a novel technique based on the dielectric response technique has received great attention. In particular, the polarization and depolarization current method (PDC), which employs the time-domain dielectric response technique, is widely used to reflect the polarization response information of both insulating oil and polymer materials in the main insulation system [8,9,10,11].

In the course of the dielectric response testing of the field transformer, it is difficult to maintain a constant temperature for the test process due to the impact of external conditions such as season, location, and weather, etc. However, studies have shown that temperature is one of the crucial factors affecting the results of the PDC test [12,13,14]. The increase in temperature promotes the response speed of polar particles (and weak polarity) inside the polymer materials, which enable them to establish polarization processes with less time duration. This characteristic allows the PDC curve of the sample with a similar insulation condition to present an obvious change rule with the rise of the test temperature. In this case, the state assessment result obtained by the PDC curve is unreliable in the case of the temperature effect is ignored. Therefore, it is of great significance to correct the temperature effect on the PDC results of the transformer polymer insulation materials.

In the present research, scholars have carried out studies of the temperature effect on PDC data. In [13], the variation law of PDC during temperature fluctuation is studied, which pointed out that there is a notable difference in the PDC measured during the thermal transient. Saha and Purkait [14] investigated the PDC curves at different temperatures to indicate that the polarization time will be shortened as the temperature increase. In response to the above research results, the master curve technique based on the time-temperature superposition theory is used as an available model to correct the temperature effect of the transformer polymer insulation materials, which provides a novel idea for related research [15,16,17,18,19,20]. However, the temperature effect is considered as the single factor affecting the PDC test results in the existing research, which ignores the fact that moisture content is also an important factor affecting the polarization data of polymer material. In other words, transformer polymer insulation materials with different moisture contents will exhibit a different response characteristic at the same temperature. Therefore, the traditional temperature correction method that considers only a single factor (temperature) could be further improved.

Based on the research of the master curve technique, this paper proposes a modified method of temperature correction of the PDC curve considering the moisture content and temperature effect. In the current work, oil-immersed polymer pressboards with different moisture contents are prepared first, and their polarization current is measured at different temperatures (45, 60, 75, and 90 °C). Then, the shift factors are extracted based on the master curve technique. Finally, a function model incorporating temperature, *mc%,* and shift factors is obtained by employing the surface fitting technique. Relying on this model, the shift factors of oil-immersed polymer pressboards with different moisture contents can be predicted, which can be used to obtain the PDC curve at various temperatures. Thus, the reported model in this paper is expected to promote the accuracy of the insulation condition assessment method of the transformer polymer materials based on the PDC technique.

## 2. The Experiment Platform

For the purpose of this study, oil-immersed polymer (cellulose) pressboards with the same degree of aging and different moisture content are prepared. The polymer pressboard has a density of 0.96 g/cm^3^ and a longitudinal tensile strength of 98 N/mm^2^. The insulating oil is No. 25 mineral insulating oil with a density of 0.8846 g/cm^3^, a breakdown voltage of 38 kV, and the kinematic viscosity is 9.652 mm^2^/s.

The pretreatment steps of the experimental samples are as follows. Firstly, the polymer pressboards and insulating oil were placed in a vacuum oven at 105 °C/50 Pa for 48 h. Then, the insulating oil and the polymer pressboards were placed in an aging tank at a ratio of 20:1 and immersed in a vacuum at 60 °C/50 Pa for 48 h. Finally, the pretreated oil-immersed polymer pressboards are obtained. Since each insulating polymer pressboard is pretreated under the same experiment conditions, it can be considered that the pretreated samples have the same initial moisture content.

In order to obtain samples with different moisture contents, it is necessary to conduct the moisture absorption on oil-immersed polymer pressboards. Firstly, three polymer pressboards were randomly selected from the pretreated samples, and the moisture contents were measured by the Karl Fischer Moisture Titrator. If the difference in the moisture contents of the three polymer pressboards is <0.3%, the average moisture content of the three is taken as the moisture content of all the polymer pressboards, recorded as *a*%. The polymer pressboards sample is placed in a precision balance to record its weight (*m*), and then it is moisture-absorbed by a humidifier until its weight reaches *M,* where *M* is calculated by the formula *m*·(1 − *a*%)/(1 − *b*%), where *b*% is the target moisture content. Finally, the insulating polymer pressboards with different moisture contents were prepared according to the above steps.

It is worth noting that the polymer pressboard is a two-phase system composed of alternating crystalline and amorphous regions. There are many gaps in the polymer amorphous region. However, if the polymer is immersed in insulating oil before moisture absorption, the molecules of insulating oil will replace the water molecules into the gaps in the amorphous area of the polymer, which makes it difficult for moisture absorption. Therefore, the moisture content mentioned in this article is mainly the moisture on the polymer surface.

The dielectric response tester (by means of DIRANA, OMICRON Co., Ltd., Klaus, Austria) is selected as the polarization current test equipment during the experiment. The device can provide a test voltage of 100 mV–200 V, and the PDC measurement range is 20–20,000 s. In this experiment, the temperature range is set to be 45–90 °C, and the step size is 15 °C. Putting the samples at the target temperature for 24 h before each measurement activities, so as to achieve the dynamic balance of moisture diffusion between the polymer pressboards and the insulating oil. The moisture content of a sample is measured. The experimental process of this paper is shown in Figure 1.

## 3. Analysis of Measurement Results

Since the variation law of current depolarization with temperature is basically consistent with the variation law of current polarization, accordingly, this paper only presented the polarization current of the samples for analysis.

In this paper, oil-immersed polymer pressboards with different moisture contents (0.81%, 1.56%, 2.62%, and 3.84%) are utilized to measure their polarization currents at 45 °C, 60 °C, 75 °C, and 90 °C, respectively. The test voltage of equipment is set to 200 V and the test duration is 5000 s. In order to measure the test data as accurately as possible, the polymer pressboards are short-circuited for 2 h before each measurement start. Afterwards, the test results are shown in Figure 2, which has been drawn in log-log scales due to it is capability of preserving the general shape of the curve and to amplify the slight changes between the different measured curves.

The test results presented that the polarization current of each sample tends to move to the upper left as the test temperature increases. This phenomenon indicates that the test temperature will generate a greater impact on the PDC curve. As the temperature of the tested oil-immersed polymer pressboards increases, the response speed and migration rate of the particles inside the samples are promoted, which makes the DC conductivity of the sample become larger. Meanwhile, the increases of the temperature accelerate the movement of polar particles and the relaxation time constant decreases, which results in a shorter time for the polarization current to reach a stable value. In addition, the balance of moisture between the insulating oil and pressboards is also affected by temperature changes, which influences the relaxation behavior to some extent.

In fact, studies have shown that the test temperature only shifts the PDC curve without changing its shape [16,17,18]. The PDC curves of the oil-immersed polymer pressboards tested at different temperatures can be shifted to coincidence by a series of the movement (horizontal and vertical). The process of moving the measured PDC curve to the reference temperature is called the construction process of the master curve, and the PDC curve shifted to the reference temperature is defined as the master curve [19,20]. The degree of movement of the curve can be expressed as the time-shift factor and the amplitude-shift factor, respectively. The expression formulas are as follows:

(1) $$\tau_{T}=\frac{t_{Tref}}{t_{T}}$$

(2) $$\alpha_{T}=\frac{h_{T}}{h_{Tref}}$$

where *T* is the test temperature, *T_ref_* is the reference temperature, *τ_T_* is the time-shift factor at temperature *T*, while *t_T_* and *t_Tref_* are the time corresponding to a certain point in the polarization current curve at *T* and *T_ref_*, respectively. Similarly, *α_T_* is the amplitude-shift factor, while *h_T_* and *h_Tref_* are the amplitude corresponding to a certain point in the polarization current curve at test temperature and the reference temperature, respectively.

In this paper, since 45 °C is regarded as the reference temperature, the polarization current curves measured at 60 °C, 75 °C, and 90 °C can be moved to coincide with the curve at the reference temperature. The movement results are shown in Figure 3.

As shown in Figure 3, the shifted curve mostly coincides with the curve of 45 °C to form a smooth curve. The time-shift factors and the amplitude-shift factors of the oil-immersed polymer pressboards with four kinds of moisture contents at different temperatures can be obtained from Equations (1) and (2). The calculation results are shown in Table 1 and Table 2, respectively.

All samples in this experiment are non-aged polymer pressboards, and the pretreatment process is carried out under the same conditions, thus, it can be considered that the samples belong to the same aging state. In terms of the calculation results of Table 1 and Table 2, it is known that the time-shift factor and amplitude-shift factor of each sample will increase with the test temperature, and this conclusion is consistent with the existing research results [17,20]. However, the authors observed that the higher the *mc%* of the sample, the greater the shift factor at the same temperature. This phenomenon indicates that *mc%* also has a significant effect on the shift factor, as well as the temperature. Therefore, the authors suggest that it is inappropriate to consider only the effect of test temperature. Further, it is necessary to comprehensively consider the effect of moisture content and temperature, so as to improve the accuracy of the temperature correction model.

## 4. Construction of Temperature Correction Model

### 4.1. Mathematical Analysis

The polarization current consists of three parts: the conductance current, the instantaneous current caused by the displacement polarization, and the absorption current cause by the relaxation polarization [8], which can be expressed as Equation (3).

(3) $$i_{p}=C_{0}U_{0}[\frac{\delta }{\varepsilon_{0}}+\varepsilon_{\infty }\delta \left(t\right)+f\left(t\right)]$$

where *C*_0_ represents the geometric capacitance of the polymer material, *U*_0_ is the amplitude of DC voltage, *δ* is the DC conductivity, *ε*_0_ represents the vacuum permittivity, *ε*_∞_ is the permittivity when sampling frequency approaches infinity, *δ*(*t*) is the Dirac delta function, which exists only at the moment of pressurization, and *f*(*t*) is the response function of the relaxation behavior.

It can be seen from Equation (3) that the DC conductivity and the relaxation behavior of the polymer material are the main factors that affect the polarization current test results. The relaxation time constant *τ*(*T*) is used to describe the rate of the dielectric relaxation behavior, and *τ*(*T*) is temperature-dependent. The “Arrhenius” equation is an empirical formula used to describe the relationship between the rate constant of a chemical reaction and temperature [21]. The relationship between *τ*(*T*) and temperature can also be described by the “Arrhenius” equation [17,20]. The equation can be written as follows:

(4) $$\tau (T)=A\cdot e^{-\frac{E_{a}}{R\cdot T}}$$

where *R* is the gas constant (*R* = 8.314 J/mol·K), *T* is the chemical reaction temperature, and *E*_a_ is the activation energy of a chemical reaction. The *A* is the pre-exponential factor, and [22] indicates that its value is related to moisture content and oxygen concentration. In addition, ref. [23] pointed out that there is a dynamic compensation effect between the pre-exponential factor and the activation energy. Therefore, activation energy is affected by moisture content, and its values can no longer be regarded as constants, but variable and related to moisture content. Equation (4) can be modified as Equation (5).

(5) $$\tau {\left(T\right)}_{i}=A_{i}\cdot e^{-\frac{E_{a}{}_{i}}{R\cdot T}}$$

where *T* represents the test temperature, *i* indicates the moisture content of the tested sample, and *τ*(*T*)*_i_* indicates the relaxation time constant corresponding to the sample with moisture content *i* at test temperature *T*. The *A_i_* and *E_ai_* represent the pre-exponential factor and activation energy of the sample with moisture content *i*, respectively.

From the time-temperature superposition theory, it is known that for the same mechanical relaxation phenomenon of polymer, the results observed at the higher temperature and shorter time are equivalent to those observed under the low temperature for a long time. In other words, the temperature only affects the rate of molecular motion without changing its reaction law. Therefore, the temperature dependence of *τ*(*T*) is mainly reflected by changing the polarizability of the polymer material, which causes the polarization current curve to shift horizontally with the temperature change. The time-shift factor can be obtained from the ratio of *τ*(*T*) at different temperatures. The expression formula is as follows:

(6) $$\tau_{iT}=\frac{\tau {\left(T_{0}\right)}_{i}}{\tau {\left(T\right)}_{i}}=\frac{A_{i}}{A_{i}}\cdot \exp[\frac{1}{R}(\frac{E_{ai}}{T}-\frac{E_{ai}}{T_{0}})]=\exp(\frac{M_{Ti}}{R})$$

where

(7) $$M_{Ti}=E_{ai}\cdot (\frac{1}{T}-\frac{1}{T_{0}})$$

Equation (7) shows that *M_Ti_* is a variable related to moisture content and temperature.

In addition, the relationship between the DC conductivity of the polymer material and the temperature also satisfies the Arrhenius equation as shown in Equation (8) [8].

(8) $$\delta_{T}=A\cdot e^{-\frac{E_{a}}{R\cdot T}}$$

where *δ_T_* is the DC conductivity of the oil-immersed polymer pressboards at the temperature *T*. Similarly, the amplitude-shift factor can also be derived from changes in *δ_T_*, and it will also be affected by *mc%* and test temperature. As a result, the amplitude-shift factor can be expressed as follows.

(9) $$\alpha_{Ti}=\frac{\delta_{Ti}}{\delta_{T_{0}i}}=\frac{A_{i}}{A_{i}}\cdot \exp[\frac{1}{R}(\frac{E_{ai}}{T_{0}}-\frac{E_{ai}}{T})]=\exp(\frac{N_{Ti}}{R})$$

Equations (7) and (9) show that the time-shift and amplitude-shift factors of the polarization current are related to the temperature and activation energy in the “Arrhenius” equation. In other words, they are all affected by temperature and *mc*%. This is consistent with the test results in the previous section. In summary, it is necessary to propose a temperature correction model of the time-domain dielectric response considering both *mc*% and temperature effects.

### 4.2. Construction of the Modified Model

In order to improve the accuracy of the temperature correction, this paper select the temperature and *mc*% as the independent variables, the time-shift factor as the dependent variable, three-dimensional coordinates are constructed and used to describe the effect of *mc*% and temperature on the time-shift factor. The fitted surface is shown in Figure 4.

Figure 4a is a three-dimensional scatter plot drawn using the time-shift factors extracted in the previous section. It can be seen from the coordinates that there is a clear relationship between the time-shift factor, temperature, and *mc*%. Its functional relationship is established by the surface fitting technique, and the results are shown in Figure 4b. The fitted surface is drawn by means of a smooth surface fitting equation, and related fitting data are shown in Table 3.

In the table, x represents the test temperature in Kelvin temperature (K), y indicates the moisture content of the sample to be tested. The goodness of fit exceeds 0.99, indicating that the fitted surface is highly matched with the sample points, which can well describe the variation of the sample.

The fitting process of the amplitude-shift factor is similar to the previous one, taking the data in Table 2 as the sample point, and the fitting result is shown in Figure 5.

It can be observed from the above results that the amplitude-shift factor also has a significant correlation with temperature and *mc%*. The relevant fitting parameters are shown in Table 4. The surface fitting goodness is as high as 0.994, which indicates that the selected fitting function is compatible with the data distribution.

The shift factors of the polarization current at diverse tested temperatures and moisture contents can be obtained by the surface fitting equations given in this section, which are defined as the prediction time-shift factor and predicted amplitude-shift factor. Then, the test curve at any temperature can be moved to the reference temperature by the predicted shift factors.

## 5. Verification Case of Temperature Correction Model

Depending on the analysis of the oil-immersed polymer pressboards with four moisture contents, this article discussed the effect of temperature and moisture content on the polarization current curve. The time-shift factor and the amplitude-shift factor are therefore extracted. Moreover, a functional model between moisture content, temperature and shift factors is established. However, the samples used for the model construction are non-aged oil-immersed polymer pressboards. This section will verify the feasibility of the proposed model with oil-immersed polymer pressboards under different insulation states (including the degree of polymerization and moisture content).

The oil-immersed polymer pressboards with different insulation states are selected and tested for polarization current curves at 45 °C, 60 °C, 75 °C, and 90 °C. Their moisture content and DP value are measured when the test is completed. Combined with the fitting equations described above, the calculation results of prediction time-shift factors of each verification sample at different temperatures are shown in Table 5.

Similarly, the prediction amplitude-shift factors can be calculated from the fitting equations in Table 4. The calculation results are shown in Table 6.

Temperature correction of the polarization current curves at different temperatures can be achieved by the above prediction shift factors. The temperature correction result of the verification samples is shown in Figure 6.

The curves under different insulation states can well coincide with the reference curve obtained by the shifting process according to the prediction shift factors. The verification test results reveal that the degree of aging does not have a significant impact on the shift factor, which indicates that the temperature correction model proposed in this article is suitable for samples with different insulation states (including the degree of polymerization and moisture content). However, due to the limited experimental conditions, the sample selected in this paper is representative, and the amount of data is slightly insufficient. The analysis of the temperature correction model using sample data of more different insulation states is a follow-up work that needs to be carried out.

## 6. Conclusions

Based on the time-temperature superposition theory, this paper explores the effect of moisture content and test temperature on the shift factor of the polarization current curve. A functional relationship model is established based on the fitting formula between the shift factor, moisture content, and the test temperature. The conclusions are drawn and elaborated as follows.

- The analysis of the shift factors of oil-immersed polymer pressboards with different moisture contents shows that the value of the shift factor is not only affected by temperature, but also by moisture content.
- A functional relationship among moisture content, temperature, and shift factors is established based on the surface fitting technique. The model can be used to calculate the shift factor at diverse temperatures and moisture contents. The results reveal that the shift factor is positively correlated with the moisture content and test temperature. The reported approach (shown in Table 3) for predicting the shift factor is established by the depth analysis of the variation law among shift factors, moisture content, and test temperature.
- The results of the verification test show that the proposed model can be applied to the temperature correction of the polarization current curve of samples with different insulation states (including moisture content and the degree of aging).

## Figures and Tables

**Figure 1:**
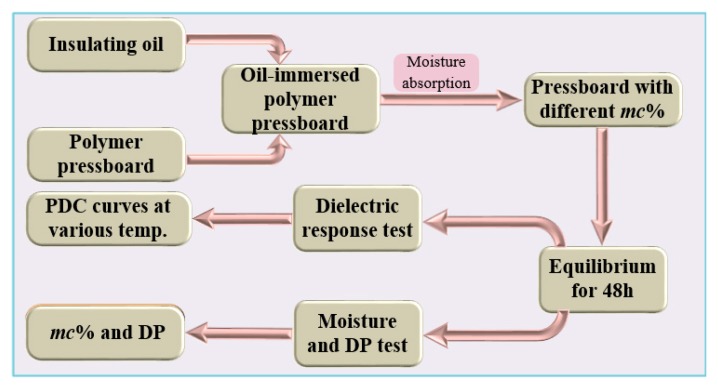
The preparation and testing process of samples.

**Figure 2:**
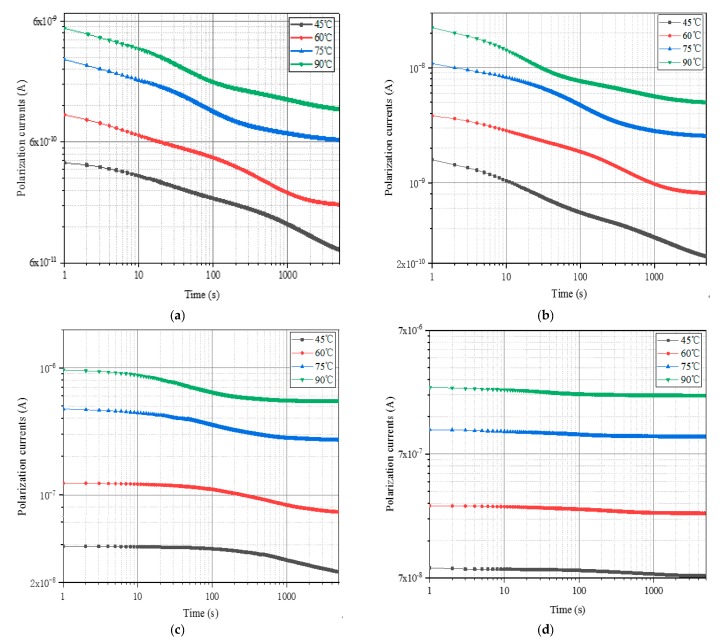
The polarization currents of unaged polymer pressboards with various moisture contents at different test temperatures. (**a**) *mc%* = 0.81%, (**b**) *mc%* = 1.56%, (**c**) *mc%* = 2.62%, (**d**) *mc%* = 3.84%.

**Figure 3:**
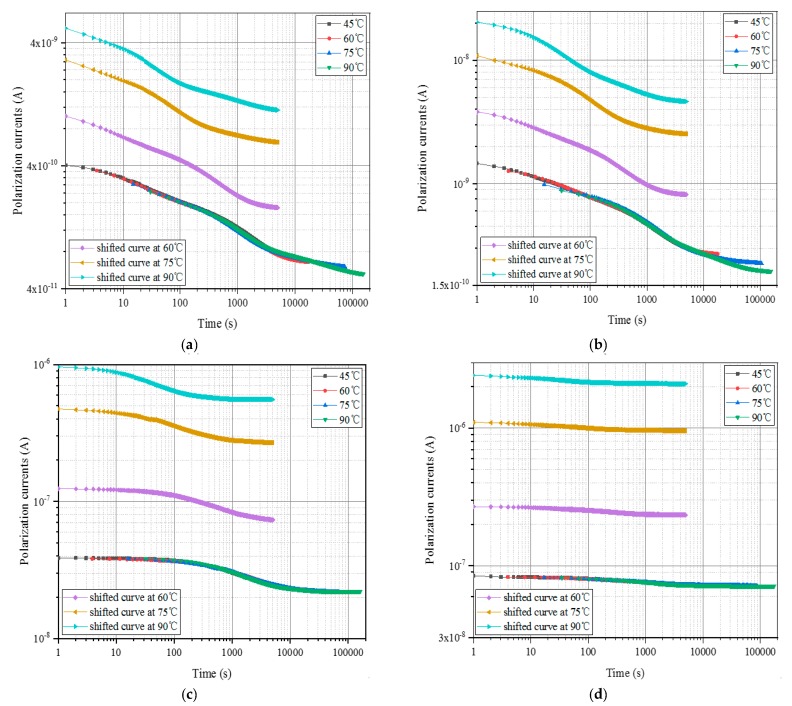
The construction results of the master curve of unaged polymer pressboards with various moisture contents at different test temperatures. (**a**) *mc*% = 0.81%, (**b**) *mc*% = 1.56%, (**c**) *mc*% = 2.62%, (**d**) *mc%* = 3.84%.

**Figure 4:**
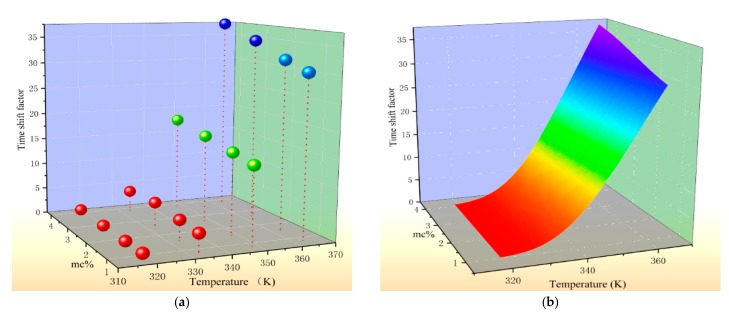
The variation law of time-shift factor. (**a**) The scatter plot of test temperature, *mc%* the and time-shift factor; (**b**) The fitting surface of the time-shift factor.

**Figure 5:**
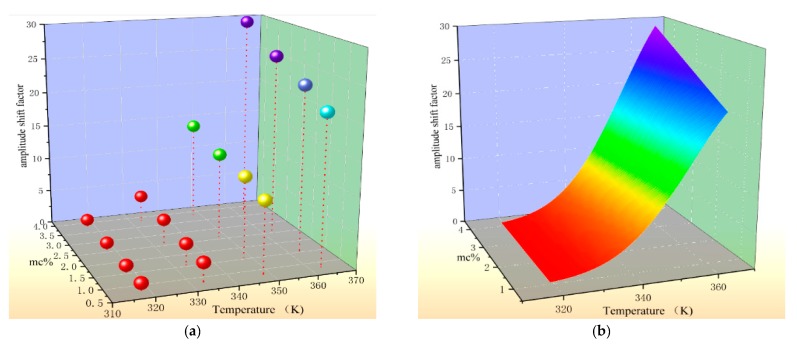
The variation law of amplitude-shift factor. (**a**) The scatter plot of test temperature, *mc*% and amplitude-shift factor; (**b**)The fitting surface of the amplitude-shift factor.

**Figure 6:**
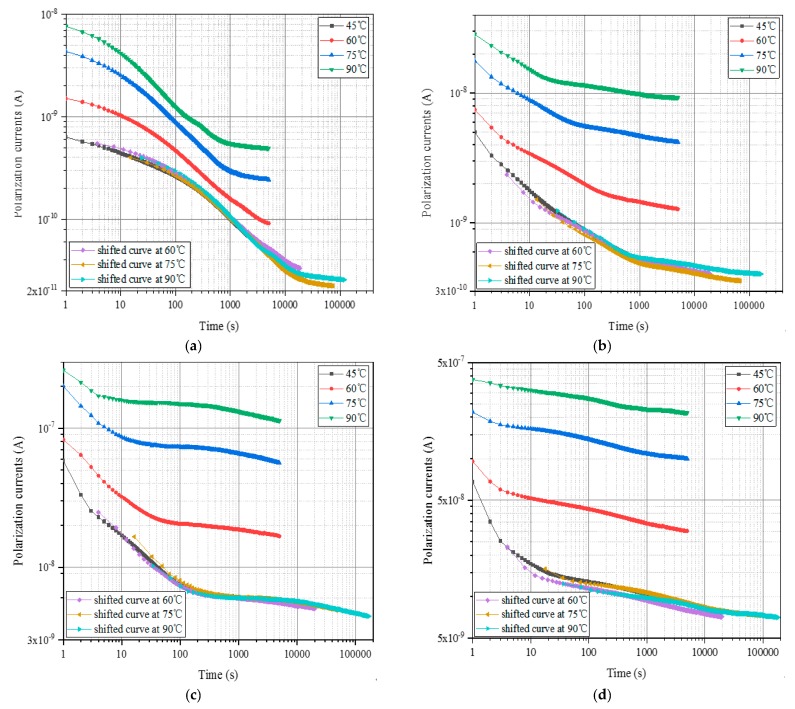
Temperature correction results of the verification samples. (**a**) DP = 795, *mc*% = 1.21%, (**b**) DP = 634, *mc*% = 1.74%, (**c**) DP = 525, *mc*% = 2.34%, (**d**) DP = 512, *mc*% = 3.52%.

**Table 1:** Calculation results of the time-shift factors at various temperatures.

*mc%*	Measured Value			
	318.15 K	333.15 K	348.15 K	363.15 K
0.81%	1.00	3.47	14.67	30.49
1.56%	1.00	3.89	15.51	32.03
2.62%	1.00	4.51	16.72	34.82
3.84%	1.00	3.93	18.01	37.39

**Table 2:** Calculation results of the amplitude-shift factors at various temperatures.

*mc%*	Measured Value			
	318.15 K	333.15 K	348.15 K	363.15 K
0.81%	1.00	2.75	10.31	21.40
1.56%	1.00	3.07	11.69	23.70
2.62%	1.00	3.49	12.38	26.08
3.84%	1.00	3.83	14.13	29.53

**Table 3:** Surface fitting equation of the time-shift factors.

$\begin{array}{ll} \tau =Z_{0} & +Z_{1}\times \exp(-\exp(-(x{-Z}_{2}{)/Z}_{3}{))+Z}_{4}\times \exp(-\exp(-(y{-Z}_{5}{)/Z}_{6}))\\ & {+Z}_{7}\times {\exp(-\exp(-(x-Z}_{2}{)/Z}_{3})-\exp(-(y{-Z}_{5}{)/Z}_{6})) \end{array}$					
*Z*_0_	0.927	*Z*_4_	−0.062	Precision	10^−15^
*Z*_1_	55.768	*Z*_5_	2.065	Degree of freedom	6
*Z*_2_	355.329	*Z*_6_	1.519	R-Square	0.997
*Z*_3_	19.884	*Z*_7_	21.712	Fit Status	succeeded

**Table 4:** Surface fitting equation of the amplitude-shift factors.

$\alpha =Z_{0}+Z_{1}\times \exp\left(-0.5\times {\left((x-Z_{2})/Z_{3}\right)}^{2}-0.5\times \left((y-Z_{4})/Z_{5}{)}^{2}\right)\right)$			
*Z*_0_	0.704	*Z*_5_	9.763
*Z*_1_	47.706	Precision	10^−15^
*Z*_2_	371.550	Degree of freedom	6
*Z*_3_	17.685	R-Square	0.994
*Z*_4_	12.495	Fit Status	succeeded

**Table 5:** Calculation results of the prediction time-shift factors.

*mc%*	DP	Calculated Value			
		318.15 K	333.15 K	348.15 K	363.15 K
1.21%	795	1.00	3.73	15.09	31.23
1.74%	634	1.00	3.85	15.69	32.51
2.34%	525	1.00	3.98	16.43	34.11
3.52%	512	1.00	4.22	17.69	36.82

**Table 6:** Calculation results of the prediction amplitude-shift factors.

*mc%*	DP	Calculated Value			
		318.15 K	333.15 K	348.15 K	363.15 K
1.21%	795	1.00	3.02	10.92	22.55
1.74%	634	1.00	3.17	11.54	23.93
2.34%	525	1.00	3.33	12.28	25.51
3.52%	512	1.00	3.66	13.73	28.63

## References

[1] Oria C., Ortiz A., Ferreño D., Carrascal I., Fernandez I. (2019). State-of-the-art review on the performance of cellulosic dielectric materials in power transformers: Mechanical response and ageing. IEEE Trans. Dielectr. Electr. Insul..

[2] Linhjell D., Lundgaard L., Gafvert U. (2007). Dielectric response of mineral oil impregnated cellulose and the impact of aging. IEEE Trans. Dielectr. Electr. Insul..

[3] Zhang Y.Y., Li Y., Zheng H.B. (2019). Microscopic reaction mechanism of the production of methanol during the thermal aging of cellulosic insulating paper. Cellulose.

[4] Liu J.F., Zheng H.B., Zhang Y.Y., Zhou T.C., Zhao J., Li J.Q., Liu J.Q., Li J.C. (2018). Comparative Investigation on the Performance of Modified System Poles and Traditional System Poles Obtained from PDC Data for Diagnosing the Ageing Condition of Transformer Polymer Insulation Materials. Polymers.

[5] Matharage Y., Liu Q., Wang Z.D. (2016). Aging assessment of kraft paper insulation through methanol in oil measurement. IEEE Trans. Dielectr. Electr. Insul..

[6] Liu J.F., Fan X.H., Zhang Y.Y., Zheng H.B. (2019). Temperature Correction on Frequency Dielectric Modulus and Activation Energy Prediction of Immersed Cellulose Insulation. IEEE Trans. Dielectr. Electr. Insul..

[7] Rodriguez-Celis E.M., Duchesne S., Jalbert J., Ryadi M. (2015). Understanding ethanol versus methanol formation from insulating paper in power transformers. Cellulose.

[8] Liu J.F., Fan X.H., Zhang Y.Y., Zheng H.B., Zhu M.Z. (2019). Quantitative evaluation for moisture content of cellulose insulation material in paper/oil system based on frequency dielectric modulus technique. Cellulose.

[9] Saha T.K., Purkait P., Muller F. (2005). Deriving an equivalent circuit of transformers insulation for understanding the dielectric response measurements. IEEE Trans. Power Deliv..

[10] Liu J.F., Fan X.H., Zhang Y.Y., Zheng H.B., Zhang C.H. (2019). Condition Prediction for Oil-immersed Cellulose Insulation in Field Transformer Using Fitting Fingerprint Database. IEEE Trans. Dielectr. Electr. Insul..

[11] Ojha S.K., Purkait P., Chakravorti S. (2016). Modeling of relaxation phenomena in transformer oil-paper insulation for understanding dielectric response measurements. IEEE Trans. Dielectr. Electr. Insul..

[12] Fofana I., Hemmatjou H., Farzaneh M. (2010). Low temperature and moisture effects on polarization and depolarization currents of oil-paper insulation. Electr. Power Syst. Res..

[13] Fofana I., Hemmatjou H., Meghnefi F. (2011). Effect of thermal transient on the polarization and depolarization current measurements. IEEE Trans. Dielectr. Electr. Insul..

[14] Saha T.K., Purkait P. (2008). Investigations of temperature effects on the dielectric response measurements of transformer oil-paper insulation system. IEEE Trans. Power Deliv..

[15] Liu X., Liao R.J., Lv Y.D., Liu J.F., Gao J., Hao J. (2015). Study on Influences and Elimination of Test Temperature on PDC Characteristic Spectroscopy of Oil-Paper Insulation System. J. Electr. Eng. Technol..

[16] Robalino D.M. Accurate temperature correction of dissipation factor data for oil-impregnated paper insulation bushings: Field experience. Proceedings of the 2011 Annual Report Conference on Electrical Insulation and Dielectric Phenomena.

[17] Yang L.J., Qi C.L., Lv Y.D., Hao J., Liao R.J., Gong C.Y., Meng F.J. (2013). Study on Influences of Thermal Aging Time and Testing Temperatures on Time-domain Dielectric Characteristics of Oil-paper Insulation. Proc. Chin. Soc. Electr. Eng..

[18] Liu J.F., Fan X.H., Zhang Y.Y., Zheng H.B., Yao H.L., Zhang C.H., Zhang Y.B., Li D.J. (2019). A novel universal approach for temperature correction on frequency domain spectroscopy curve of transformer polymer insulation. Polymers.

[19] Zhang Y.Y., Liu J.F., Zheng H.B., Wang K. (2018). Feasibility of a universal approach for temperature correction in frequency domain spectroscopy of transformer insulation. IEEE Trans. Dielectr. Electr. Insul..

[20] Ma Z.Q., Liao R.J., Hao J., Zhao X.P., Wang Y.L., Yang L.J. (2014). Influence of temperature on polarization and depolarization current of oil-paper insulation. Trans. China Electrotech. Soc..

[21] Verma H.C., Baral A., Pradhan A.K., Chakravorti S. (2017). A method to estimate activation energy of power transformer insulation using time domain spectroscopy data. IEEE Trans. Dielectr. Electr. Insul..

[22] Teymouri A., Vahidi B. (2019). Estimation of power transformer remaining life from activation energy and pre-exponential factor in the Arrhenius equation. Cellulose.

[23] Du R.L., Wu K., Xu D.A., Chao C.Y., Zhang L., Du X.D. (2016). A modified Arrhenius equation to predict the reaction rate constant of Anyuan pulverized-coal pyrolysis at different heating rates. Fuel Process. Technol..

